# Carcinosarcoma of the Urinary Bladder: A Case Report

**DOI:** 10.1100/tsw.2005.35

**Published:** 2005-04-05

**Authors:** Limci Gupta, Jacqueline Elder

**Affiliations:** Histopathology Department, Countess of Chester Hospital NHS Foundation Trust, Chester, Cheshire, CH2 1UL, UK

**Keywords:** carcinosarcoma, rare tumour, rhabdomyosarcoma, transitional cell carcinoma, immunostaining, sarcomatoid TCC

## Abstract

Carcinosarcomas are rare tumours containing both malignant mesenchymal and epithelial elements. This reports presents a 70-year-old man with carcinosarcoma of the urinary bladder, which is proven histologically.

## CASE REPORT

A 70-year-old man had dysuria and urgency for micturition for more than 5 years off and on. He was investigated repeatedly with cystoscopy and biopsies were taken that were reported as cystitis cystica. No malignancy was noted histologically. The symptoms continued and an ultrasound and magnetic resonance imaging (MRI) of the renal tract were performed, which revealed a T3 bladder tumour with no evidence of lymphadenopathy. Transurethral resection of bladder (TURB) was performed and biopsies taken that showed a high-grade rhabdomyosarcoma. He was then treated with radical prostatocystectomy and an ileal conduit.

The resection specimen showed a large 100- × 85- × 55-mm polypoid tumour mass on the left anterolateral and posterior surface of the bladder. The rest of the bladder wall was nodular and ulcerated. Microscopically, the polypoid tumour tissue showed diffusely infiltrating malignant cells arranged in sheets and, in places, in an alveolar pattern. These cells were spindle shaped and epithelioid with large hyperchromatic nuclei. Numerous rhabdomyoblasts and bizarre multinucleated cells were seen with a background of loose connective tissue. Abundant mitotic figures were present, some of which were abnormal. Necrosis and haemorrhage were also present. The appearances were those of a high-grade rhabdomyosarcoma.

Sections from the nodular area showed poorly differentiated, solid infiltrating transitional cell carcinoma. Carcinoma *in situ* was also noted overlying the sarcomatous element. Sarcoma and carcinoma components intermingled in one focus, therefore, it was regarded as carcinosarcoma. There was an incidental adenocarcinoma of prostate, Gleason Grade 3 + 3 = 6.

Immunostaining was performed. The areas of transitional cell carcinoma stained positive for both keratin and epithelial membrane antigen. The sarcomatous component stained positive for vimentin, desmin, and myoglobin. This, therefore, confirmed the diagnosis of carcinosarcoma.

## DISCUSSION

This 70-year-old man was first thought to have alveolar rhabdomyosarcoma of urinary bladder on TURB. Rhabdomyosarcoma is very rare at this age and is the most frequent tumour of the bladder in childhood occurring usually under the age of 15 years. In the review by Bostwick, sarcoma accounted for 2.7% of all primary bladder neoplasms[8]. It is extremely rare in adults and often presents with haematuria and bladder neck obstruction.

The older age and the presenting features in this case would probably refute the diagnosis of a pure rhabdomyosarcoma and favour the possibility of a combined tumour or a poorly differentiated (sarcomatoid) transitional cell carcinoma. Extensive sampling was done from the resection specimen.

In view of separate areas of the tumour revealing a pure sarcomatous component without dual staining for vimentin and epithelial markers, the appearances favour a carcinosarcoma. Adjacent and overlying carcinoma *in situ* with associated rests of invasive carcinoma present within the sarcoma again suggest a carcinosarcoma rather than a form of dedifferentiated transitional cell carcinoma.

Carcinosarcomas are rare tumours containing both malignant mesenchymal and epithelial elements[1]. Meyer[4] classified the carcinosarcomas into three histogenetic groups. One of them is a collision tumour in which carcinoma and sarcoma coincidentally arise in proximity and then invade each other. The two other groups include combination tumours in which both the carcinomatous and sarcomatous components arise from a pluripotential cell and composition tumours in which both components derive from the same tissue concomitantly. Neoplastic transformation may occur in two distinct tissues or secondary sarcomatous change may develop in the stroma of a carcinoma. Willis[5] has limited the term carcinosarcoma more rigidly by excluding the group of collision tumours.

Carcinosarcomas are usually highly aggressive and the 5-year survival rate is approximately 20%. Although these tumours are resistant to radiation therapy, most investigators agree that the best outlook for cure includes a combination of surgery and irradiation[6].
